# The Mori Cable: A Novel Anti-Twist, Dual-Output Cable to Optimize Left Bundle Branch Area Pacing

**DOI:** 10.7759/cureus.114507

**Published:** 2026-08-13

**Authors:** Kenji Mori, Takahiro Kamihara, Atsuya Shimizu

**Affiliations:** 1 Department of Clinical Engineering, National Center for Geriatrics and Gerontology, Obu, JPN; 2 Department of Cardiology, National Center for Geriatrics and Gerontology, Obu, JPN

**Keywords:** anti-twist, cable, dual-output, left bundle branch area pacing, mori cable

## Abstract

Left bundle branch area pacing (LBBAP) requires multiple rapid clockwise rotations of the pacing lead for deep septal deployment. Standard connection cables frequently twist and entangle during this maneuver, necessitating repeated disconnections that interrupt real-time electrogram (EGM) monitoring and increase the risk of lead displacement. To overcome these limitations, we developed the Mori Cable, a novel pacing cable featuring a 360-degree free-rotating coaxial alligator clip and a bifurcated dual-output interface. This technical report describes the device's structural architecture and mechanical performance. The independent rotation mechanism successfully prevents cable twisting while maintaining continuous electrical contact. Concurrently, the dual-output configuration allows simultaneous connection to the external stimulator and the electrophysiology system, enabling uninterrupted, real-time EGM monitoring during lead advancement.

## Introduction

Left bundle branch area pacing (LBBAP) has rapidly emerged as a physiological pacing modality that directly engages the cardiac conduction system to maintain physiological ventricular activation [1]. To achieve stable deep septal deployment, the pacing lead must be actively driven into the interventricular septum via multiple rapid clockwise rotations [2, 3]. During this maneuver, continuous intracardiac electrogram (EGM) monitoring is clinically essential; real-time observation of QRS morphology transitions, current of injury, and left bundle branch potentials guides penetration depth and alerts the operator to potential septal perforation.

However, conventional connection cables are rigidly fixed to both the lead pin and the recording apparatus. Consequently, continuous lead rotation causes severe cable twisting and entanglement, forcing operators to repeatedly disconnect the cable. These frequent disconnections create critical informational "blind spots" during active lead advancement and inherently increase the risk of accidental lead dislodgement. To overcome these practical limitations of standard hardware, we designed and developed the Mori Cable.

## Technical report

Device design and mechanism

**Figure 1:**
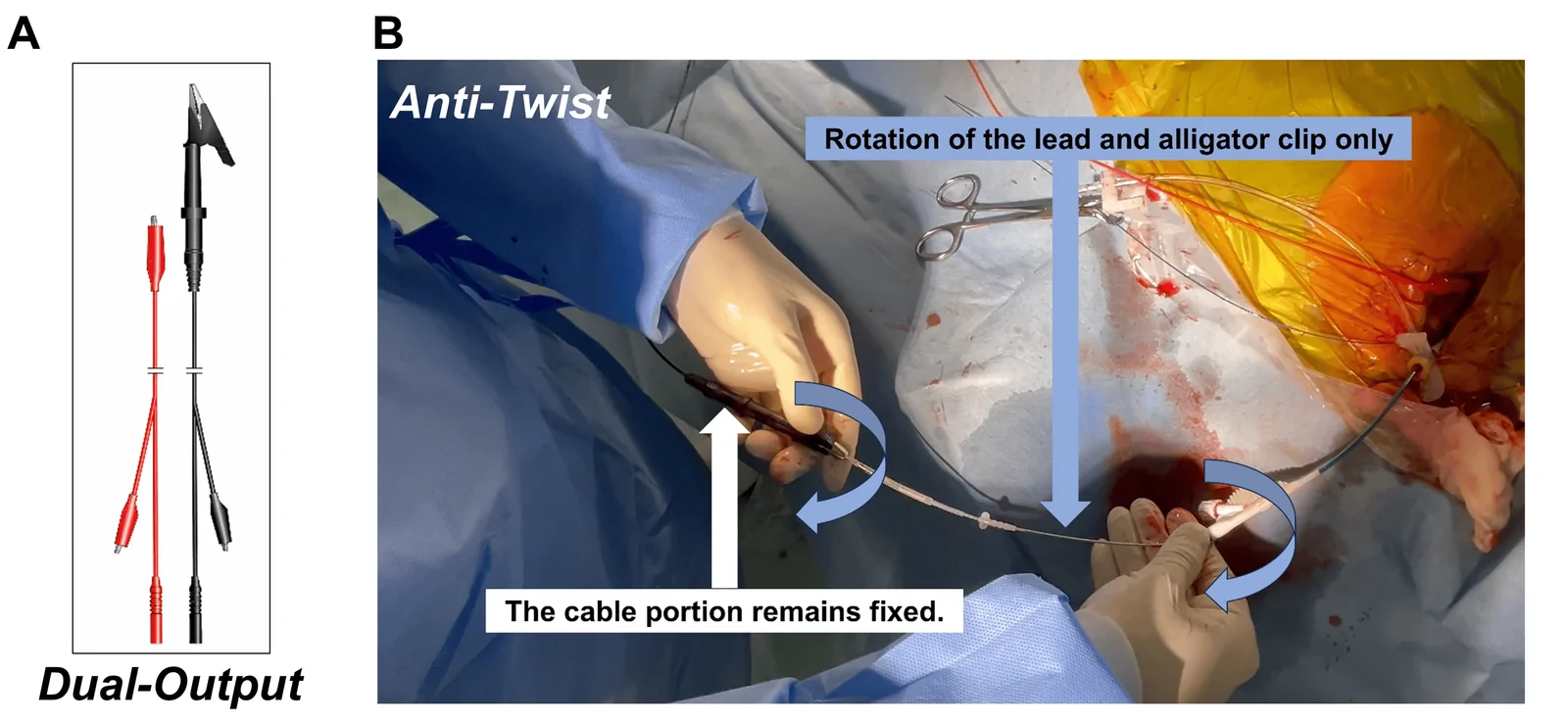
Structural design and anti-twist mechanism of the Mori cable. (A) Dual-output terminal interface: The proximal terminal features a bifurcated connector splitting into two parallel pairs of standard 2-mm pin connectors, allowing simultaneous connection to an external pacing stimulator and an electrophysiology (EP) recording system. (B) Anti-twist mechanism: The distal terminal incorporates a custom 360-degree free-rotating coaxial alligator clip. As clockwise rotation is applied to the lead pin (indicated by the blue circular arrow), the clip head rotates freely while the main cable body remains completely stationary and untwisted (white arrow), preserving mechanical stability.

The core innovation of the Mori Cable (currently under commercial development in collaboration with Taisho Biomed Instruments Co., Ltd., Osaka, Japan) lies in its mechanical isolation and dual-output terminal architecture (Figure 1A). The device integrates a specialized 360-degree free-rotating coaxial alligator clip at the distal terminal and a bifurcated, parallel connector at the proximal end. Despite the dual-line design, the terminal alligator clip is mechanically decoupled from the main cable body. This design allows the clip head to spin smoothly and infinitely in full alignment with the rotating lead pin, without transmitting any torsional torque or rotational force back into the cable lines (Figure 1B).

Operational workflow

**Figure 2:**
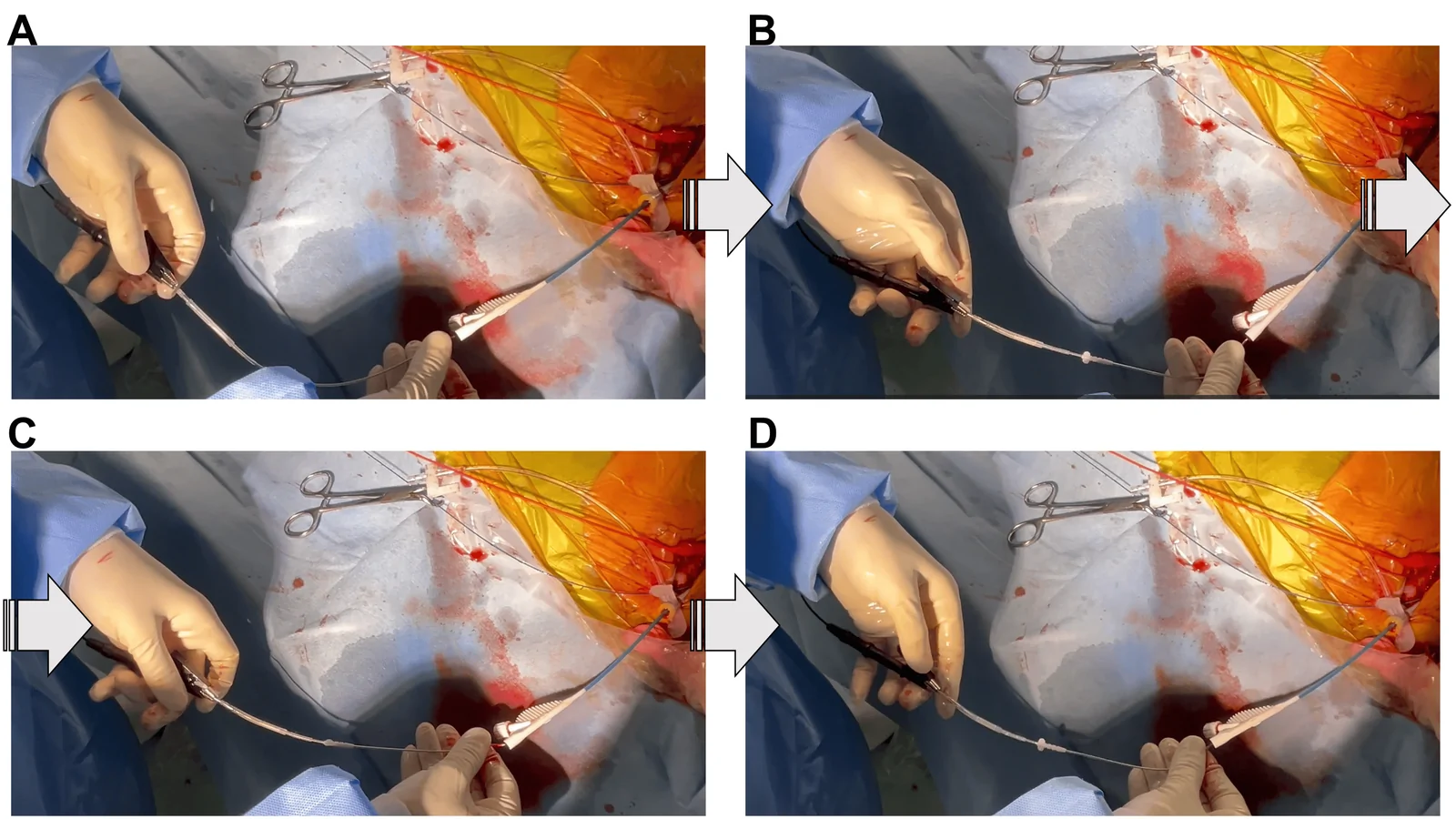
Intraoperative demonstration of seamless lead rotation. (A–D) Sequential images of lead advancement: This chronological sequence illustrates the continuous clockwise rotation of the pacing lead for deep septal penetration. The Mori Cable maintains a stable and uninterrupted electrical connection to the lead pin throughout the entire maneuver. Note that the proximal main cable remains slack and untwisted despite multiple rotations, ensuring mechanical stability and minimizing the risk of macroscopic lead displacement.

During the LBBAP procedure, the rotating alligator clip maintains a secure, uninterrupted electrical grip on the lead pin as the operator applies clockwise torque to deploy the lead deep into the interventricular septum. While the lead and clip head rotate in unison, the primary cable portion remains completely stable, fixed, and free of tension (Figures 2A-2D).

**Figure 3:**
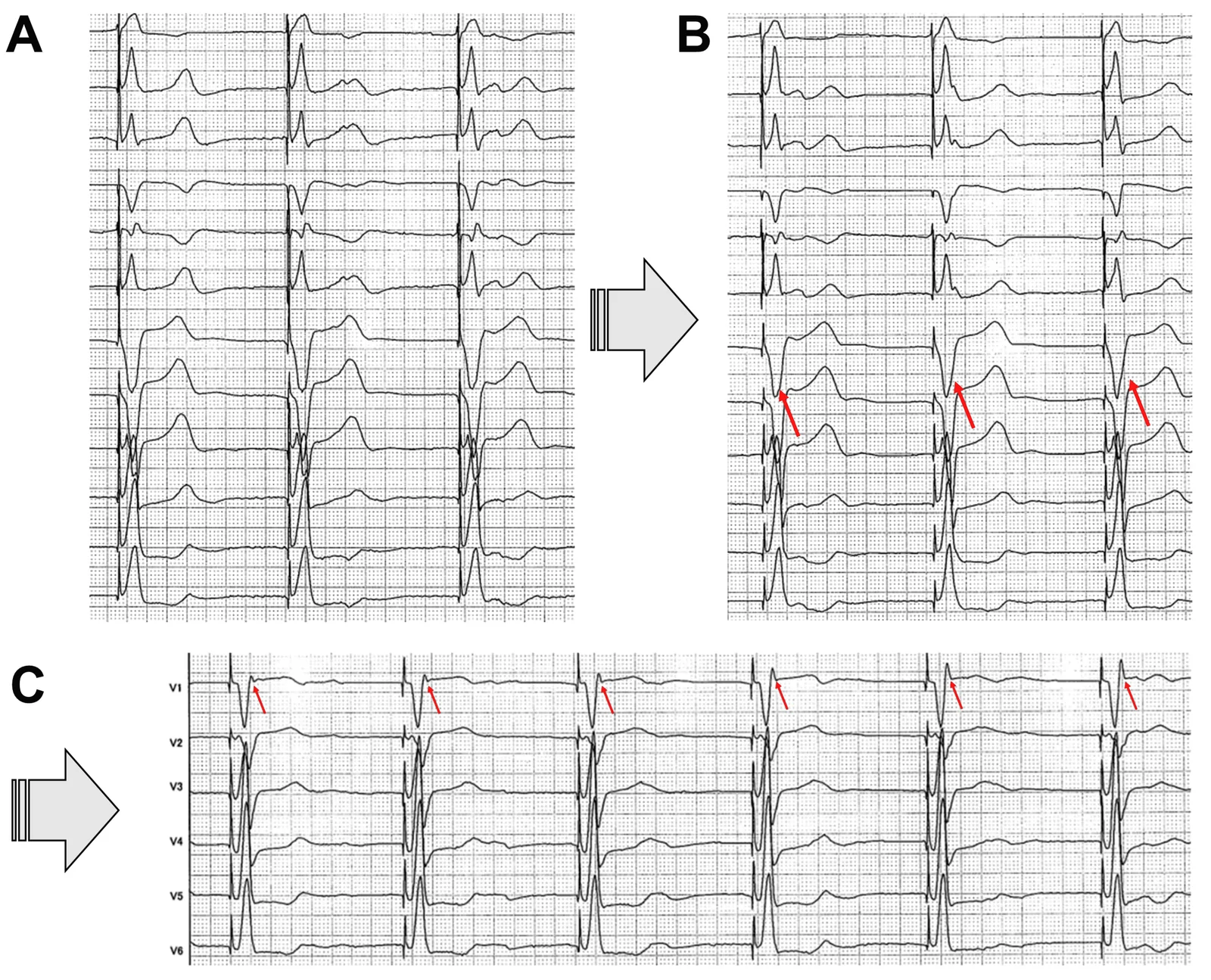
Real-time electrogram monitoring and left bundle branch (LBB) capture representative surface ECG and intracardiac electrogram recordings. (A) Baseline electrocardiogram prior to lead advancement; (B, C) Continuous monitoring facilitated by the dual-output interface during lead penetration. Red arrows indicate critical morphological changes and the sharpening of the LBB potential. The Mori Cable’s integrated architecture allows the physician to observe the transition from non-selective to selective LBB area pacing in real-time without informational "blind spots" caused by cable disconnections.

Simultaneously, the proximal bifurcated interface permits parallel, concurrent connections to both the external pacemaker stimulator and the electrophysiology recording system (Figures 3A-3C). This eliminates the traditional clinical need for line switching or manual cable disconnections during active lead advancement.

## Discussion

The Mori Cable directly resolves the mechanical and electrophysiological bottlenecks associated with lead deployment in LBBAP. LBBAP has emerged as a physiological pacing strategy with proven long-term efficacy and stable performance in pacing-dependent patients [1]. Since its early descriptions highlighting its feasibility and safety in atrioventricular block [2], prospective evaluations have confirmed its superior electrophysiologic and echocardiographic characteristics compared to conventional right ventricular pacing [3]. These outcomes contrast with His bundle pacing, which, despite its physiological promise, continues to face challenges regarding long-term capture stability and clinical outcomes, as highlighted in a recent large-scale multicenter prospective study in Japan by Yanagisawa et al. [4].

It should be emphasized that this article serves primarily as a technical description and proof-of-concept report of a novel device architecture. Quantitative evaluations, including mechanical torque resistance, electrical signal-to-noise ratio comparisons, and prospective clinical outcome measurements, were beyond the scope of this initial technical report and will be conducted in subsequent prospective studies.

While the initial clinical application demonstrated excellent operational feasibility without cable twisting, objective mechanical evaluations (such as rotational cycle limits and torsional resistance) and electrical impedance measurements remain to be formally quantified in future engineering bench tests. We explicitly acknowledge that the present report is intended as a technical description and proof-of-concept of a novel device. Because the cable was adopted as our standard interface from our very first clinical case, quantitative comparative metrics, such as procedure duration, signal continuity parameters, and comparative success rates, were not established against standard cables and warrant formal prospective validation. Specific technical evaluations, including rotational cycle endurance, tensile and torsional resistance, electrical impedance stability, and direct signal quality comparison with standard cables, represent critical next steps that will be evaluated in subsequent engineering and clinical investigations. Nevertheless, the Mori Cable may help overcome several procedural challenges associated with LBBAP that have been highlighted in previous reports [5-10]. Its unique dual-output design enables simultaneous pacing and continuous EGM monitoring while allowing unrestricted mechanical rotation of the lead, thereby minimizing interruptions in electrophysiological monitoring and facilitating safe and smooth lead manipulation.

Limitations

This technical report has several inherent limitations. First, because the Mori Cable was routinely implemented at our institution from our initial LBBAP case due to its high structural feasibility and procedural convenience, no comparative clinical data against conventional pacing cables or quantitative bench-testing data (e.g., rotational endurance cycles, tensile strength, or electrical impedance) were collected. Second, device durability under repeated sterilization, long-term connector wear, and broader regulatory considerations for widespread commercial use remain to be fully evaluated in collaboration with the manufacturer. Finally, prospective, randomized clinical studies are required to confirm whether the proposed structural and operational advantages translate into statistically significant improvements in procedural duration, lead stability, and safety outcomes.

## Conclusions

The Mori Cable represents a novel engineering concept designed to address cable twisting and monitoring interruptions during LBBAP lead placement. By combining a 360-degree free-rotating coaxial clip with a dual-output interface, the device demonstrates technical feasibility for continuous EGM monitoring and unhindered lead rotation. Future prospective clinical trials and objective bench evaluations are needed to formally validate its clinical performance and long-term utility.

## References

[1] Yao Y, Sun M, Sheng Y (2025). Long-term follow-up of left bundle branch area pacing in pacing-dependent patients and normal cardiac function. Pacing Clin Electrophysiol.

[2] Li X, Li H, Ma W (2019). Permanent left bundle branch area pacing for atrioventricular block: feasibility, safety, and acute effect. Heart Rhythm.

[3] Vijayaraman P, Subzposh FA, Naperkowski A (2019). Prospective evaluation of feasibility and electrophysiologic and echocardiographic characteristics of left bundle branch area pacing. Heart Rhythm.

[4] Yanagisawa S, Kato H, Kanzaki Y (2026). Long-term prognosis and outcomes in patients undergoing his bundle pacing implantation-a multicenter, prospective observational study in Japan. Circ J.

[5] Burri H, Zupan Mežnar A, Witte KK (2026). Conduction system pacing: what emerging evidence and what indications?. Europace.

[6] Montenegro Sá F, Castro A, Pereira S, Gavina C (2026). Conduction system pacing using a rotatable connector enabling continuous pacing during lumenless lead deployment: a case report. Eur Heart J Case Rep.

[7] Dyrbuś M, Stempfel S, Valiton V, Tolppanen H, Burri H (2026). Characteristics of paced waveforms and current of injury prior to septal perforation during left bundle branch area pacing. Europace.

[8] Clark J, Holmes L, Williams OL, Easson L, Brooks V (2026). Unipolar diathermy applied via the terminal pin to release an entrapped LBBAP lead helix: a case report. Eur Heart J Case Rep.

[9] Wakasa S, Yoshiyama T, Hirayama S, Fukuda K, Yanagishita T, Hayashi Y, Fukuda D (2026). Feasibility and efficacy of left bundle branch area pacing guided by modified chest lead 1. J Electrocardiol.

[10] Dell'Era G, Loumaye L, Marinaccio L (2026). Multicenter experience with a helix locking tool for stylet-driven lead fixation in left bundle branch area pacing. Pacing Clin Electrophysiol.

